# Research Progress in Phase–Structure Evolution and Electro-magnetic–Wave Absorption Performance of Aluminosilicate Mineral–Based Ceramics

**DOI:** 10.3390/nano16191245

**Published:** 2026-10-01

**Authors:** Zhongyi Bai, Qichen Zhang, Song Li, Chunjiang Li, Biao Zhao, Mengjie Wang, Xuwen Li, Rong Wang, Xiaoqin Guo

**Affiliations:** 1 Henan Key Laboratory of Aeronautical Materials and Application Technology, School of Materials Science and Engineering, Zhengzhou University of Aeronautics, Zhengzhou 450046, China; 2 School of Materials Science and Engineering, Zhengzhou University, Zhengzhou 450001, China

**Keywords:** aluminosilicate, molten salt synthesis, phase-structure evolution, absorption performance

## Abstract

High-temperature electromagnetic functional ceramics require matrix phases, such as alumina and mullite, that combine resistance to high temperatures and oxidation with low density. Aluminosilicates derived from natural minerals or solid wastes can reduce material costs and introduce additional electromagnetic responses through associated iron-, carbon-, and alkali-metal-containing components. However, fully exploiting these advantages requires coordinated control of activation, separation, molten salt recovery, and heat treatment. This review systematically examines six precursor types—kaolin, perlite, sillimanite, red mud, fly ash, and coal gangue—with particular emphasis on their phase characteristics, phase-structure evolution and regulation mechanisms in molten-salt environments, porous-structure construction, and electromagnetic functionalization. Current evidence indicates that kaolin and sillimanite primarily follow mullitization pathways, whereas mullitization of perlite requires exogenous aluminum components. Under defined carbon-source and atmospheric conditions, fly ash, coal gangue, and red mud can form multiphase composites containing carbon, iron-based phases, and aluminosilicates. This review synthesizes the relationships among precursor composition, phase-structure evolution pathways, and interfacial structure, and identifies high-temperature in situ electromagnetic characterization, improved oxidation stability, and scalable preparation as key priorities for future research. Perlite is discussed more briefly because quantitative electromagnetic-wave absorption data for perlite-derived ceramics remain limited.

## 1. Introduction

Electromagnetic wave (EMW) absorbing materials are widely used in military stealth, electromagnetic compatibility protection, and electromagnetic-pollution control in 5G/6G communication base stations [1,2]. As aircraft speeds increase and the power density of electronic devices rises rapidly, their required service-temperature range has expanded from room temperature to 600–1200 °C. Conventional magnetic absorbers, conductive polymers, and carbon-based absorbing agents generally cannot satisfy service requirements in oxygen-containing high-temperature environments. Ceramic-based EMW absorbers, which offer inherent resistance to high temperatures and oxidation together with low density, have therefore become a major focus of high-temperature EMW absorption research [3,4].

Alumina (Al_2_O_3_) and mullite (3Al_2_O_3_·2SiO_2_) possess excellent high-temperature stability and structural robustness [5], making them important matrix or skeleton phases for high-temperature absorbing composites. Because both phases have limited intrinsic dielectric loss, additional attenuation pathways must be introduced through defect regulation, heterointerface construction, or conductive and magnetic second phases. Qi et al. [6] discussed microstructural design strategies for activating electromagnetic functions in ceramic absorbers, whereas Li et al. [7] reviewed lightweight and heat-resistant ceramic absorbing materials. At present, Al_2_O_3_/mullite ceramics are commonly synthesized through high-purity chemical-reagent routes. In a representative process, metallurgical-grade alumina is extracted from bauxite by the Bayer process, mixed with high-purity SiO_2_ at a stoichiometric ratio, and converted to the desired phase through high-temperature solid-state synthesis. Although this route is well established, it requires substantial energy and generates large quantities of red mud by-product, which is inconsistent with sustainable-development objectives [8]. In addition, studies on the activation of non-bauxite aluminum sources include kaolin “sintering-leaching” [9], high-sulfur bauxite “suspension calcination-alkali leaching” [10], fly ash–red mud co-calcination [11], as well as direct solid-state sintering of waste sand/bauxite into mullite ceramics [12] and hydro-chemical leaching routes [13]. The entire preparation system remains constrained by high energy consumption, process complexity, difficult impurity removal, and inadequate phase controllability. Further research is therefore needed to achieve precise phase-structure regulation and controllable fabrication of low-cost, green, high-performance high-temperature absorbing ceramic matrices through integrated “optimized activation–separation–heat treatment.”

**Figure 1:**
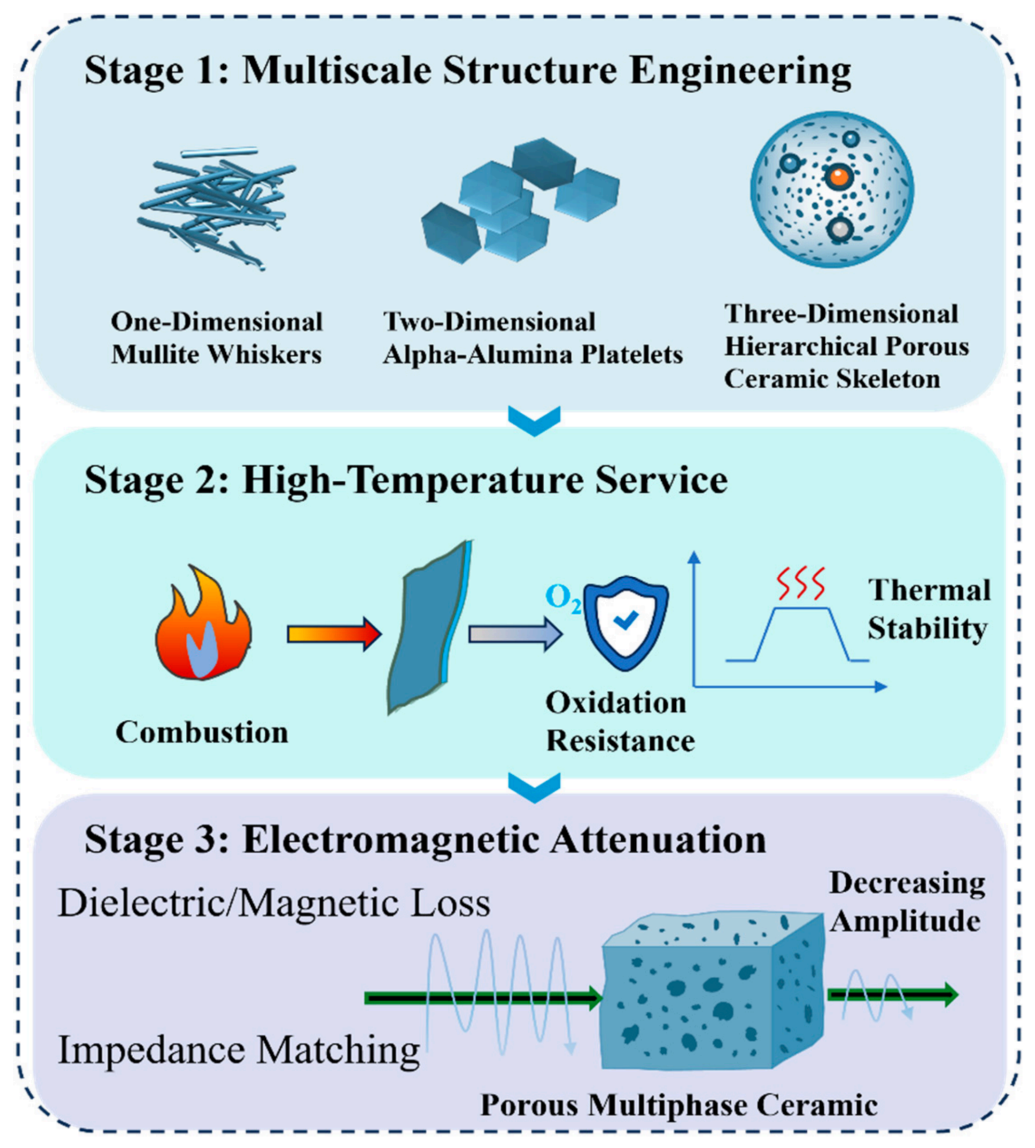
Cross-scale design roadmap for converting typical aluminosilicate precursors into EMW absorbing ceramics.

Within this context, natural aluminosilicate minerals (kaolin, perlite, and sillimanite) and industrial aluminum-silicon-rich solid wastes (fly ash, coal gangue, and red mud) have attracted increasing attention as precursors for electromagnetic functional ceramics. Their compositional diversity and complex impurity systems provide alternative routes for the low-cost fabrication of functional ceramic materials [14,15]. This review systematically examines activation methods, phase-structure evolution pathways, and EMW absorption characteristics of ceramics derived from these six precursor types, and evaluates the relationships among composition, phase evolution, and interfacial structure to guide future development. Figure 1 summarizes the overall framework. The fundamental features of aluminosilicate-derived ceramics are first outlined, followed by phase-structure regulation strategies involving one-dimensional mullite whiskers, two-dimensional α–Al_2_O_3_ platelets, and three-dimensional hierarchical/porous architectures. Phase reconstruction and the role of impurity chemistry in electromagnetic response are then discussed, with emphasis on dielectric and magnetic losses and on dielectric and oxidation stability at high temperatures. Finally, future research should prioritize high-temperature in situ electromagnetic characterization, improved oxidation stability, and scalable preparation processes. Because only a few studies report quantitative EMW absorption data for perlite-derived ceramics, perlite is discussed more briefly than kaolin and sillimanite and is used primarily to illustrate constraints associated with silicon-rich amorphous phases and alkali-related stability. The six precursors define the primary scope of this review; other materials are included only when required to explain general mechanisms and are identified as model or analogous systems rather than direct precursor derivatives.

## 2. Fundamentals of Aluminosilicate Mineral-Based Electromagnetic Functional Ceramics

### 2.1. Electromagnetic Functional Advantages of Alumina and Mullite Matrices

**α–Al_2_O_3_:** From the perspective of crystal chemistry, O^2–^ in α–Al_2_O_3_ adopts a hexagonal close-packed arrangement, with Al^3+^ occupying two-thirds of the octahedral voids to form [AlO_6_] coordination polyhedra. This compact arrangement provides α–Al_2_O_3_ with a melting point of about 2050 °C, a Mohs hardness of about 9.0, and high chemical inertness [16,17,18,19]. These features allow the phase assemblage and structural integrity of α–Al_2_O_3_ to be maintained under high-temperature, corrosive, and mechanically demanding conditions. Nevertheless, its highly ordered and symmetric lattice contains relatively few intrinsically responsive dipoles. Pure α–Al_2_O_3_ therefore typically exhibits low dielectric loss, and its dielectric behavior must be improved through morphology engineering, which increases interface density and polarization sites, or defect engineering, which introduces oxygen vacancies, unsaturated coordination sites, and other dipole-relaxation centers [20,21]. Furthermore, coupling alumina with conductive components such as carbon materials or SiC, or with magnetic components such as Fe_3_O_4_ or ferrite, can increase dielectric loss through space-charge accumulation at heterointerfaces. This behavior is generally attributed to the Maxwell–Wagner interfacial polarization effect caused by space-charge accumulation across heterogeneous phase boundaries and microstructural interfaces [22,23].

**Mullite:** Mullite can be represented as Al_2_(Al_2+2x_Si_2−2x_)O_10−x_, with a solid-solution composition typically corresponding to x = 0.17–0.59 [24]. From the perspective of crystal chemistry, mullite consists of [AlO_6_] octahedral chains interconnected by AlO_4_/SiO_4_ tetrahedra, with disordered Al/Si distribution and associated oxygen vacancies [24,25]. This structure provides a low intrinsic dielectric constant of approximately 6.5–6.8 for dense mullite [20] and a low coefficient of thermal expansion [25], while introducing porosity or whisker architecture can significantly reduce the effective dielectric constant down to below 3.0 [20]; conductive or impurity phases are generally introduced to increase dielectric loss. The chain-like structure also favors growth along the *c*–axis over the *a*– and *b*–axes, allowing single-crystal mullite whiskers with aspect ratios of about 20–100 to form in molten salt media.

Overall, α–Al_2_O_3_ and mullite form thermally stable ceramic skeletons, but their pure phases generally behave as low-loss dielectrics. Microwave attenuation can be improved by introducing defect-mediated polarization, heterointerfaces, and conductive or magnetic second phases. Under defined reducing conditions, some fly ash, coal gangue, and red mud systems can generate carbon, Fe_3_O_4_, Fe, or iron-silicon phases. Nevertheless, the resulting phase assemblage [26,27,28,29,30] and functional performance are strongly dependent on feedstock composition, added components, atmosphere, and heat-treatment conditions.

### 2.2. Phase Evolution Pathways of Aluminosilicate Precursors

**Figure 2:**
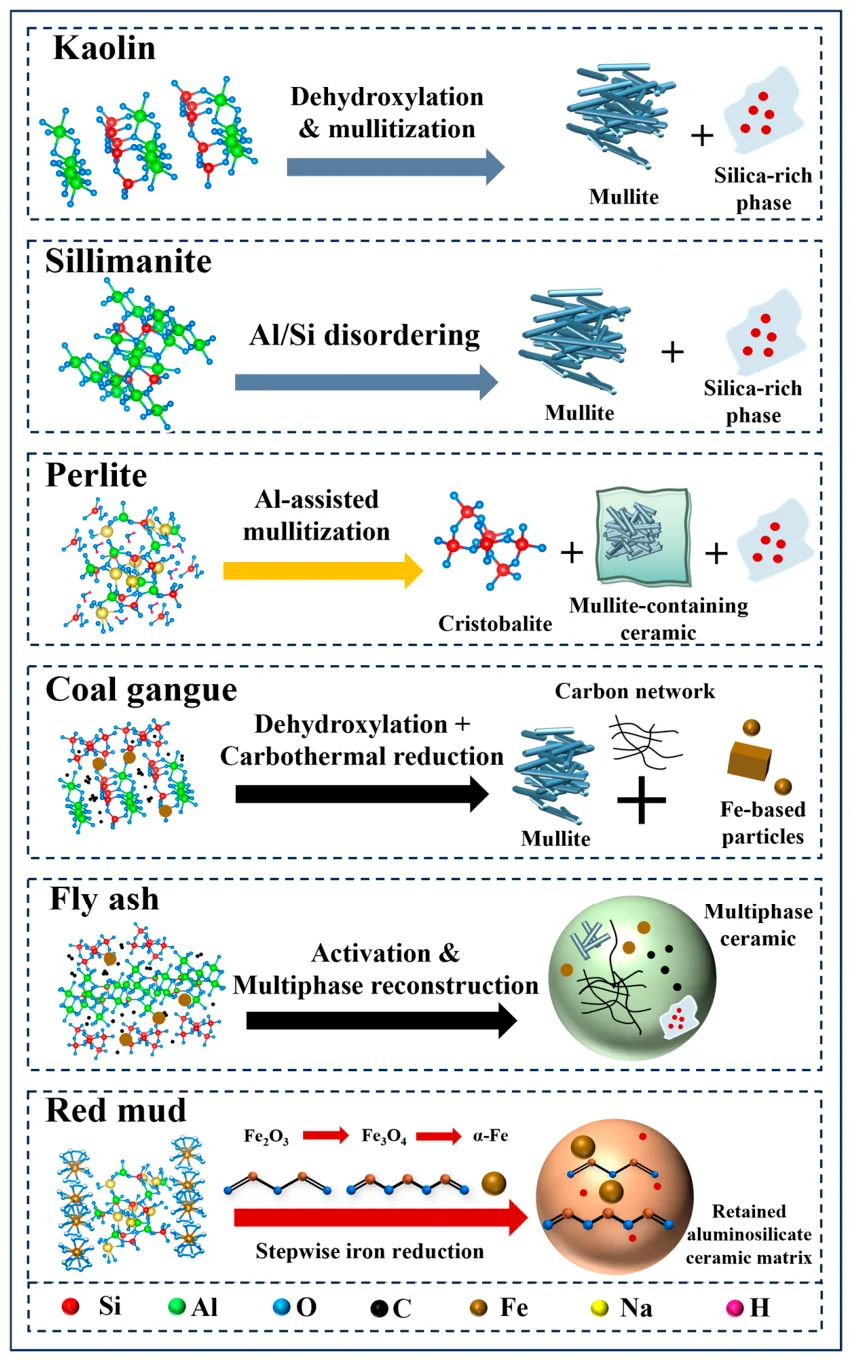
Crystal structures, representative properties, and phase transformation pathways of six aluminosilicate precursors (kaolin, sillimanite, perlite, coal gangue, fly ash, and red mud) for the preparation of microwave-absorbing ceramics.

The six aluminosilicate precursors all contain SiO_2_ and Al_2_O_3_ as major components, but their phase structures range from fully amorphous perlite to crystalline sillimanite and kaolin and to mixed-phase fly ash, coal gangue, and red mud. These intrinsic differences govern the activation conditions required to convert natural minerals or solid wastes into reactive ceramic precursors and consequently produce distinct high-temperature reconstruction pathways [31,32,33,34]. The main pathways are: (1) classical mullitization of kaolin and sillimanite; (2) inert-lattice depolymerization and multiphase synergy during carbothermal reduction in fly ash and coal gangue; (3) delayed transformation of perlite owing to its silicon-rich amorphous phase; and (4) stepwise reduction in endogenous iron oxides accompanied by multiphase coexistence in red mud. Figure 2 compares the principal features and reconstruction behavior of each precursor according to its dominant phase-transformation mechanism.

Differences among these four pathways originate from precursor structure, particularly structural order, impurity type, and Al/Si stoichiometric ratio. Kaolin and sillimanite both follow mullitization pathways, but the underlying mechanisms differ markedly. Kaolin first undergoes dehydroxylation to form metakaolinite [35]; removal of structural hydroxyl groups disrupts the ordered layered structure and generates a metastable aluminosilicate phase with increased chemical activity. Formation of a single mullite phase generally requires either an additional aluminum source or an atomically mixed precursor because the initial Al/Si ratio does not fully satisfy the stoichiometric requirement of mullite [25,36]. Moreover, mullite formed from kaolin-based precursors develops through a well-defined structural evolution during mullitization [37], and naturally dense kaolin provides little inherent porosity and contains no intrinsic magnetic component [20,38]. Sillimanite shares the [AlO_6_] octahedral skeleton of mullite, and its transformation starts with reduced tetrahedral Al/Si order and partial component separation, followed by discontinuous conversion into mullite and a silicon-rich glass phase. The transformation temperature is affected by detection sensitivity, particle size, impurity content, holding time, and the surrounding chemical environment, while complete conversion usually requires temperatures above 1500 °C [24,39,40]. Fly ash and coal gangue instead undergo carbothermal reduction coupled with multiphase reconstruction. Stable mullite and quartz in fly ash must first be chemically activated to break Al–O–Si bonds, whereas the glassy phase may dissolve or react more readily, producing nonuniform reaction rates among particles. Residual carbon can form conductive networks, and iron-containing phases may contribute magnetic loss [26,27,41,42,43,44]. In coal gangue, undecomposed clay minerals coexist with carbonaceous components, and phase transformation involves kaolinite dehydroxylation and crystallization together with carbothermal reduction of iron oxides. High-carbon batches can also act as in situ reducing agents: the fixed carbon in coal gangue provides a reducing environment that supports carbothermal reduction [45], while the additive-catalysed calcination and desilication behaviour of the same feedstock has been characterized in detail [46]. A similar in situ reduction role has been reported for red-mud/coal-gangue mixtures [30]. Perlite and red mud follow non-equilibrium or impurity-dominated pathways. Perlite is a silicon-rich amorphous glass with an Al_2_O_3_/SiO_2_ ratio well below the stoichiometric requirement for mullite. It expands on rapid heating at 900–1200 °C [47]; at higher temperatures, viscous sintering and cristobalite precipitation dominate unless exogenous aluminum is introduced [38,48,49,50,51,52]. In red mud, Fe_2_O_3_ exceeds 40%, and phase evolution is controlled by stepwise iron-oxide reduction, producing coexisting Fe_3_O_4_, α–Fe, and sodium aluminosilicates [28,29].

### 2.3. Relationship Between Phase-Structure Regulation and Electromagnetic Functionalization of Aluminosilicate-Derived Ceramics

Al_2_O_3_ and mullite can serve as ceramic skeletons for phase-structure and interfacial design. Two-dimensional platelets and one-dimensional whiskers can alter interface density, pore architecture, and EMW propagation paths, but these structures alone do not produce strong dielectric loss. The extent of interface polarization at Al_2_O_3_/mullite heterointerfaces is affected by the conductivity difference between the phases, defect states, interfacial bonding, and interface quality. Therefore, combining a low-dielectric ceramic skeleton with a controlled amount of loss phase represents a testable design strategy [53,54,55,56].

Electromagnetic functionalization in aluminosilicate-derived ceramics can be considered at three structural scales. (1) At the atomic level, oxygen vacancies, coordination changes, and heterovalent doping can form defect dipoles that affect dielectric relaxation [21,25]. (2) At the microscopic level, platelet orientation, whisker interlocking, and interfacial distribution affect crack propagation and the local electric field. Conduction or interfacial-polarization losses become evident only when conductive components or significant differences in conductivity are introduced [39]. (3) At the macroscopic level, porosity reduces the effective dielectric constant and improves impedance compatibility; however, this effect is constrained by the porosity, pore size, connectivity, and matrix parameters [3,57,58,59,60,61,62]. These factors frequently interact with each other, among which defect concentrations are affected by synthesis conditions and impurities [26,27,28,29,30].

Overall, Al_2_O_3_ and mullite provide ceramic skeletons suitable for service at high temperatures, but their intrinsically low-loss behavior prevents them from functioning independently as efficient absorbing media. On the basis of structural order, Al/Si stoichiometry, and impurity type, the six aluminosilicate precursors can be classified into four high-temperature phase-evolution pathways: mullitization, multiphase co-reconstruction, delayed phase transformation, and multiphase coexistence. More broadly, these pathways can be grouped into mullitization, carbothermal-reduction reconstruction, and impurity-dominated evolution. Although phase-structure regulation alters polarization-site density and interfacial states across atomic, microscopic, and macroscopic scales, one-dimensional whiskers, two-dimensional lamellar crystals, and three-dimensional porous skeletons do not intrinsically generate strong dielectric or magnetic loss [63].

## 3. Phase-Structure Regulation of Aluminosilicate-Derived Ceramic Systems

Following precursor activation, controlling the morphology and spatial distribution of product crystals at different scales is critical for electromagnetic functionalization. Once reactive Al^3+^ and Si^4+^ species are generated, regulation of the morphology and spatial distribution of the target ceramic matrix, such as Al_2_O_3_ or mullite, and the in situ-derived impurity phases remains an important challenge. Crystal nucleation, anisotropic growth, particle aggregation, and phase separation can occur simultaneously, and their relative rates determine the final architecture [64,65,66,67]. Such architectural control is also what makes functional integration possible, as illustrated by mullite insulation tiles into which microwave absorption has been integrated alongside thermal insulation [68]. This section compares the similarities and differences in structural regulation of different precursors at three scales: one-dimensional mullite whiskers, two-dimensional α–Al_2_O_3_ platelets, and three-dimensional hierarchical or porous frameworks. MSS is considered a common method for morphology regulation. This scale-based comparison connects precursor reactivity with the final spatial distributions of the matrix and functional phases.

### 3.1. Controlled Growth of One-Dimensional Mullite Whiskers

**(1) MSS growth mechanism of mullite whiskers**

**Figure 3:**
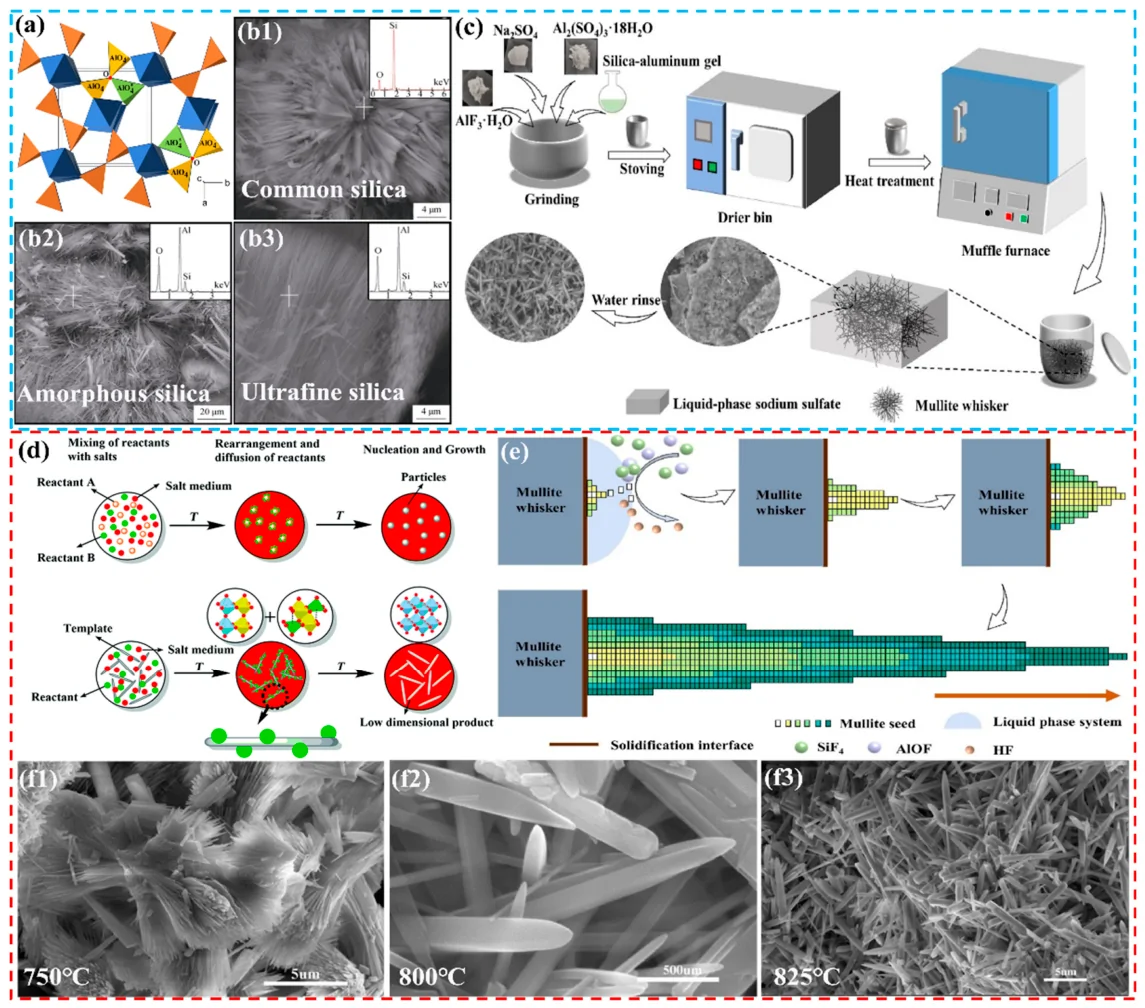
Mullite crystal structure, growth mechanism, and process parameter effects on microstructure. (**a**) Crystal structure of mullite, where orange and blue polyhedrons represent AlO_4_ tetrahedrons and AlO_6_ octahedrons, respectively. Reprinted with permission from ref. [37]. Copyright 2024 Elsevier Ltd. and Techna Group S.r.l. (**b1**–**b3**) SEM images and EDS results of mullite whiskers synthesized using different silicon sources. Reprinted with permission from ref. [75]. Copyright 2015 Springer-Verlag Berlin Heidelberg. (**c**) Preparation process of mullite whiskers. Reprinted with permission from ref. [77]. Copyright 2022 Elsevier Ltd. and Techna Group S.r.l. (**d**) Schematic showing the mechanisms of the conventional MSS and TMSS routes. Reprinted with permission from ref. [70,72]. Copyright 2020 Elsevier Ltd. and the Royal Society of Chemistry. (**e**) Scheme of the vapor-liquid-solid (V-L-S) growth mechanism of mullite whiskers using the AlF_3_·3H_2_O-assisted Na_2_SO_4_ molten-salt method; (**f1**–**f3**) SEM images of mullite whiskers at different sintering temperatures. Reprinted with permission from ref. [77]. Copyright 2022 Elsevier Ltd. and Techna Group S.r.l.

MSS methods have been reviewed systematically elsewhere and are therefore not repeated here [69]. Because of its chain-like crystal architecture, mullite has an intrinsic tendency to grow preferentially along the *c*–axis. Specifically, [AlO_6_] octahedral chains share edges along the *c*–axis, whereas growth along the *a*– and *b*–axes requires bridging by AlO_4_ and SiO_4_ tetrahedra and therefore occurs at a substantially lower rate. Figure 3a schematically illustrates the mullite crystal structure. In MSS environments, the low viscosity and high diffusivity of the molten salt, together with the choice of silicon and aluminum sources (Figure 3(b1–b3)), further strengthen this anisotropic growth tendency; across the broader family of precursor-derived routes, the same source-medium-additive interplay governs the growth habit and phase assemblage of the resulting mullite [70,71,72,73,74,75,76]. The preparation route for mullite whiskers is summarized in Figure 3c. Gupta [70] described the fundamental MSS process as a “dissolution-precipitation” mechanism (Figure 3d): precursors partially dissolve in a low-melting inorganic salt melt and release reactive ions or ionic groups, nucleation occurs once the concentration exceeds the critical supersaturation of the desired crystal phase, and dissolved species subsequently deposit on the nuclei to drive directional crystal growth. Sulfate systems, including Na_2_SO_4_, K_2_SO_4_, and their mixed salts, are commonly used to prepare oxide ceramic powders by MSS [70,71]. Figure 3(e,f1–f3) illustrates the typical process flow and microstructural evolution during one-dimensional mullite-whisker formation from silicon-aluminum precursors in a Na_2_SO_4_ molten-salt system via this “dissolution-precipitation” mechanism [77].

**(2) Key factors influencing the morphology of mullite whiskers**

**Figure 4:**
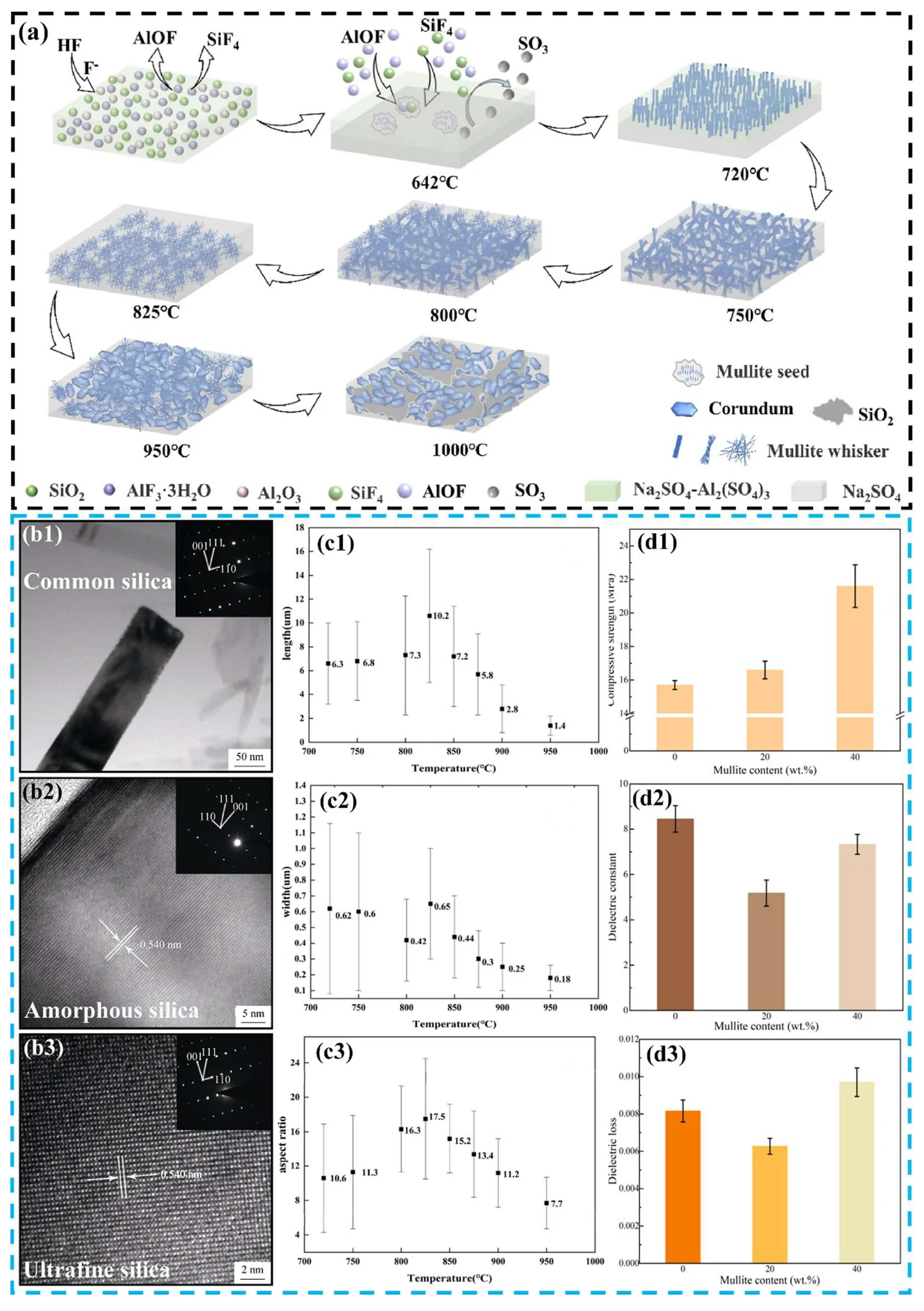
Process parameter effects on mullite whisker morphology, mechanical properties, and dielectric properties. (**a**) Morphological evolution model of mullite whiskers at different sintering temperatures. Reprinted with permission from ref. [77]. Copyright 2022 Elsevier Ltd. and Techna Group S.r.l. High-resolution transmission electron microscope (HRTEM) images of mullite whiskers prepared with (**b1**) common silica, (**b2**) amorphous silica, (**b3**) ultrafine silica. Reprinted with permission from ref. [75]. Copyright 2015 Springer-Verlag Berlin Heidelberg. (**c1**–**c3**) Length, width, and aspect ratio of mullite whiskers as functions of the firing temperature. Reprinted with permission from ref. [77]. Copyright 2022 Elsevier Ltd. and Techna Group S.r.l. The compressive strength and dielectric properties of the composite ceramics at 1 MHz (**d1**) compressive strength, (**d2**) dielectric constant and (**d3**) dielectric loss. Reprinted with permission from ref. [42]. Copyright 2022 Elsevier B.V.

Mullite-whisker morphology is controlled by starting-material composition, catalyst type and dosage, synthesis temperature and holding time, molten-salt medium, mixing scale, and precursor impurities. Adjusting these parameters allows effective control of whisker size, aspect ratio, and morphology [39,42,77]. Figure 4a illustrates representative process effects. Tripathi [39], for example, varied the total Al_2_O_3_ content from 57.60 to 79.16 wt% and sintered the materials at 1500–1575 °C; silicon-rich formulations mainly produced needle-like mullite, whereas aluminum-rich formulations generated more non-needle-like or flaky morphologies. Gao et al. synthesized highly monodispersed mullite whiskers with lengths of approximately 1 μm in Na_2_SO_4_ molten salt at 900 °C, entirely derived from fly ash via Na_2_CO_3_ activation and sulfuric acid leaching [42]. Effects of silica characteristics on the microstructure of mullite whiskers [75], as shown in Figure 4(b1–b3). Chen et al. used industrial waste silica-aluminum gel [77] as the precursor and introduced AlF_3_·3H_2_O into an Al_2_(SO_4_)_3_–Na_2_SO_4_ system. The products exhibited relatively high crystallinity and aspect ratios, while further temperature increases promoted corundum formation. These findings show that appropriately activated solid wastes can serve as precursors for molten-salt whisker synthesis (Figure 4(c1–c3)). Direct MSS preparation of mullite whiskers from other precursor classes remains rare apart from fly ash [42] and waste silica-alumina gel [77]. Gao et al. [42] also reported favorable dielectric properties for fly ash-derived mullite whiskers intended for electronic packaging (Figure 4(d1–d3)). In fly ash, Al_2_O_3_ occurs mainly in stable mullite grains whose dissolution rate is substantially lower than that of amorphous SiO_2_, causing local fluctuations in the Al/Si ratio [42]. After kaolin is dehydroxylated to metakaolinite, Al and Si are mixed relatively uniformly at the submicron scale; therefore, more uniform dissolution in molten salt may be expected, although this remains to be verified experimentally. The amorphous character of perlite may enhance dissolution, but its high SiO_2_ content reduces the effective Al concentration in the melt. No experimental studies have reported whisker growth from red mud or sillimanite under MSS conditions. Fly ash is one of the six selected precursors, whereas waste silica-alumina gel is included only as an additional industrial-waste comparison. Performance implication: mullite whiskers primarily act as low-loss, high-aspect-ratio dielectric skeletons; strong attenuation generally requires carbon, magnetic, or other loss phases [26,30,78,79,80,81], as illustrated by the mullite-based composites in Table S1.

### 3.2. Controlled Growth of Two-Dimensional α–Al_2_O_3_ Platelets

Most platelet-growth studies cited here used reagent-grade or model alumina rather than the six precursors. These studies are considered because Ti^4+^ impurities in fly ash and perlite may act via the same anisotropic-growth mechanisms, but they do not directly establish platelet formation from those precursors.

**(1) Principles of anisotropic growth of α–Al_2_O_3_ platelets**

**Figure 5:**
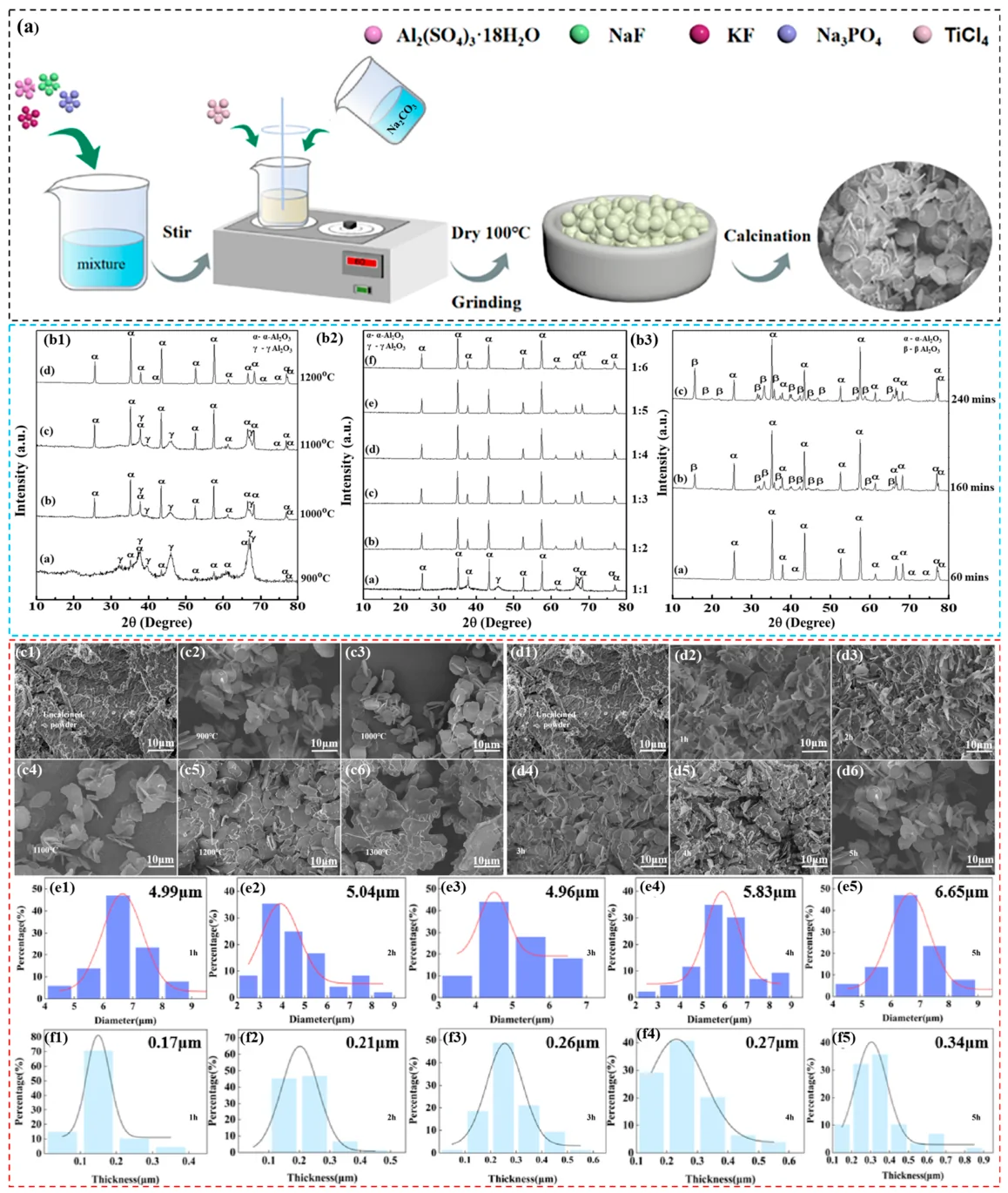
Process parameter effects on α-Al_2_O_3_ platelet phase, morphology, particle size, and (**f1**–**f5**) thickness distribution. (**a**) Schematic illustration of the synthesis of α–Al_2_O_3_ platelets via the molten-salt route. Reprinted with permission from ref. [90]. Copyright 2025 Elsevier Ltd. and Techna Group S.r.l. Effects of (**b1**) different calcination temperatures (Al/Na molar ratio of 1:2, t = 1 h), (**b2**) different Al/Na molar ratios (T = 1200 °C, t = 1 h), and (**b3**) different holding times (Al/Na molar ratio of 1:2, T = 1200 °C) on the phases of the γ–Al_2_O_3_ + Na_2_SO_4_ powder products. Reprinted with permission from ref. [89]. Copyright 2022 Elsevier Ltd. and Techna Group S.r.l. SEM morphology of (**c1**–**c6**) calcined alumina powders at different calcination temperatures, (**d1**–**d6**) alumina powders calcined at 900 °C under different holding times. (**e1**–**e5**) Particle size and (**f1**–**f5**) thickness distribution of alumina powders calcined at 900 °C under different holding times. Reprinted with permission from ref. [90]. Copyright 2025 Elsevier Ltd. and Techna Group S.r.l.

The crystal planes of corundum-structured α–Al_2_O_3_ differ markedly in atomic arrangement and surface energy. The (0001) basal plane, or c-plane, exposes Al^3+^ as the outermost surface ion and therefore has high surface polarity. Anisotropic platelet growth arises mainly because growth along the edge plane is much faster than growth normal to the basal plane. This anisotropy can be promoted by additives such as mineralizers, Ti^4+^ ions, or components that generate an intergranular liquid phase during crystal growth [82,83,84,85,86,87,88,89,90]. Figure 5a illustrates the molten-salt route used to prepare α–Al_2_O_3_ platelets. During growth, these additives preferentially adsorb on the basal plane, suppressing growth normal to this plane and directing crystal development laterally to form two-dimensional platelets. Zhu et al. [86] showed that PO_4_^3–^ supplied by Na_3_PO_4_ preferentially adsorbed on the α–Al_2_O_3_ (0001) basal plane in a sulfate molten-salt system, thereby inhibiting axial growth and producing thin sheet-like α–Al_2_O_3_. Ti^4+^ supplied as TiOSO_4_ instead promoted thicker hexagonal platelets [86]. Chang et al. compared α–Al_2_O_3_ morphologies produced in K_2_SO_4_, Na_2_SO_4_, KCl, and NaCl under identical conditions by SEM [87] and found that regular hexagonal lamellar crystals formed only in sulfate melts [88]. Using Al_2_(SO_4_)_3_ and γ–Al_2_O_3_ as aluminum sources, the study produced α–Al_2_O_3_ single-crystal sheets with diameters from ~1 μm to >20 μm and thicknesses from ~0.1 μm to 1.3 μm.

**(2) Key Factors influencing the morphology of α–Al_2_O_3_ platelets**

The precursor phase, mineralizer type, additives such as Ti^4+^ and SiO_2_, and the thermal schedule jointly control crystal-plane exposure and radial/axial growth competition in α–Al_2_O_3_, thereby determining platelet morphology. These variables primarily alter the surface energy and growth rate of individual crystal planes, promoting selective lateral growth and controlled platelet formation [89,90]. Sikder et al. [89] evaluated calcination temperature, holding time, and molten salt/precursor ratio. Increasing the calcination temperature from 900 °C to 1200 °C increased platelet diameter from about 1 μm to about 8 μm and thickness from about 200 nm to about 1 μm [89]. Calcination temperature had the strongest effect on phase formation and platelet size, the Al/Na ratio mainly controlled the aspect ratio, and prolonged soaking (240 min) caused platelet overlapping and aggregation [89]. Temperature-dependent morphology regulation is closely related to the γ → α phase transformation (Figure 5(b1–b3)). Between 900 and 1100 °C, γ–Al_2_O_3_ and α–Al_2_O_3_ coexist, and residual γ phase appears as amorphous aggregates that interfere with uniform anisotropic platelet growth. At 1200 °C, disappearance of the characteristic γ-phase diffraction peaks coincides with increased phase purity and formation of regular hexagonal platelets, helping define the appropriate temperature window for morphology control. The molten salt/precursor ratio also changes dissolution-precipitation kinetics and platelet morphology. Liu et al. [90] synthesized α–Al_2_O_3_ platelets in a NaF–KF molten-salt system at 900 °C and systematically evaluated calcination temperature (Figure 5(c1–c3)), holding time (Figure 5(d1–f5)), molten salt ratio, Na_3_PO_4_ addition, and TiCl_4_ addition. Extending the holding time at 900 °C barely changed the platelet thickness (≈ 0.26 μm) but gradually coarsened the platelets, with the average diameter remaining at ≈ 4.9–5.0 μm during the first 3 h and then increasing to ≈ 5.83 μm at 4 h and ≈ 6.65 μm at 5 h, the narrowest size distribution and the most uniform hexagonal platelets being obtained after 3 h (Figure 5(e1–f5)). An appropriate TiCl_4_ dosage promoted regular platelet formation, whereas excessive Ti^4+^ caused agglomeration and morphological deterioration [90]. Excess dopants may alter nucleation density, generate segregated phases, or reduce adsorption selectivity on specific crystal planes. This dopant-assisted anisotropic-growth concept is relevant to mineral-derived systems because naturally occurring Ti^4+^ in fly ash and perlite may similarly regulate crystal-plane growth during MSS. Performance implication: α–Al_2_O_3_ platelets mainly alter interface density and orientation rather than providing strong intrinsic absorption; their electromagnetic contribution depends on conductivity contrast with a second loss phase (Table S1).

### 3.3. Design of Three-Dimensional Hierarchical/Porous Structures

Three-dimensional networks formed from one-dimensional whiskers and spatially oriented arrangements of two-dimensional lamellar crystals can markedly increase interfacial density. Nevertheless, the effective dielectric constant of a dense single-phase ceramic may remain relatively high, thereby resulting in pronounced reflection of incident EMWs at the material surface. By constructing a three-dimensional hierarchical assembly with a connected porous skeleton, the effective dielectric constant can be lowered to improve impedance compatibility. Multiple reflections and scattering within the channels can further extend the propagation and dissipation paths of EMWs and increase attenuation [91,92,93,94]. The discussion below focuses on the pore-formation mechanism and strategies for constructing three-dimensional hierarchical or porous architectures.

**(1) Mechanisms of pore-structure formation**

Pores in ceramic skeletons may form through gas-release expansion, carbon-template removal, partial sintering, or retention of interparticle voids. Perlite, for example, releases bound water and expands during heating, with the resulting pore architecture controlled by particle size, water state, and heating rate [47,49]. Carbon, which is naturally present in coal gangue and fly ash or intentionally incorporated, can simultaneously serve as a reducing agent, a precursor for conductive loss networks (such as in situ SiC and graphitic carbon), and a sacrificial pore-forming agent. The allocation of carbon among these multifunctional roles is largely governed by the carbothermal reduction conditions, sintering atmosphere, and thermal profile [26,81]. Idrissi et al. [38] reported that, in a kaolin/perlite porous ceramic membrane sintered at 950–1100 °C, packing gaps between expanded perlite particles and between metakaolin particles were retained under partial-sintering conditions, producing an open network with pore sizes of 1–10 μm. Kaolin and sillimanite themselves do not provide directly inherited macroscopic connected porosity; consequently, porous skeletons usually require pore-forming agents, foaming, or controlled particle packing. Chen et al. [40], reinforced mullite ceramic foams via vacuum infiltration of different sillimanite-group mineral slurries (andalusite, sillimanite, and kyanite) into porous preforms, systematically comparing the effects of mineral decomposition and secondary mullitization on phase evolution, mechanical strength, and thermal shock resistance.

Overall, the pore architecture of the aluminosilicate mineral-based ceramic skeleton is formed primarily via gas escape expansion, carbonaceous oxidation pore formation, particle packing gap retention, and pore-forming agent introduction. Most existing studies are limited to describing a single mechanism. Systematic studies are still limited regarding the synergy among multiple mechanisms, quantitative control of pore parameters (pore-size distribution and open/closed porosity), their correlation with EMW absorption performance, and the stability of pore architectures during service at high temperatures.

**(2) Three-dimensional hierarchical/porous structure construction strategies**

**Figure 6:**
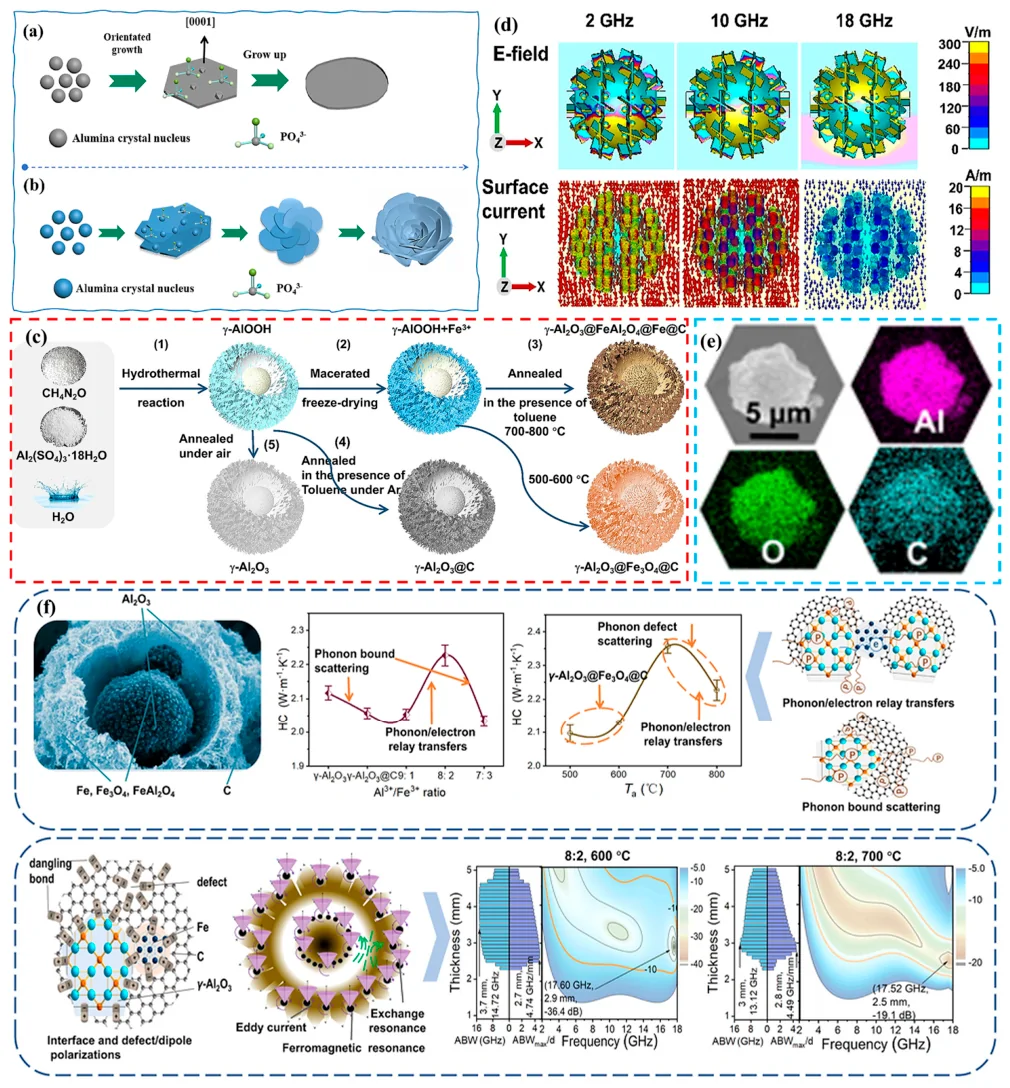
Multistage structural design and EMW absorption of Al_2_O_3_-based composites. (**a**,**b**) Growth mechanism of alumina crystal flower. Reprinted with permission from ref. [95]. Copyright 2025 Elsevier Ltd. and Techna Group S.r.l. (**c**) Synthesis and evolution routes of γ–Al_2_O_3_-based YSMSs; (**d**) Dielectric-loss mechanism based on *E*-field and surface-current distributions in the γ–Al_2_O_3_@FeAl_2_O_4_@Fe@C YSM simulated by the CST Studio Suite software; (**e**) YSMS precursor after annealing treatment; (**f**) γ–Al_2_O_3_-based yolk-shell microspheres synergistically couple magnetic/dielectric dual-loss mechanisms with phonon/electron thermal carriers, enabling collaborative microwave absorption and heat conduction. Reprinted with permission from ref. [96]. Copyright 2024 Royal Society of Chemistry.

Constructing three-dimensional hierarchical assemblies at the single-grain or grain scale offers a promising route to combine electromagnetic and thermal functions. Three-dimensional alumina crystal flowers produced by molten-salt synthesis are representative examples in which two-dimensional lamellae self-assemble into hierarchical structures [95,96]. Anisotropic growth can be driven by anion-specific adsorption. Lu et al. [95] showed that calcination temperature was the dominant parameter controlling crystal-flower morphology: single-phase α–Al_2_O_3_ was maintained from 900 to 1100 °C, while crystal-flower size increased progressively with temperature. At 1200 °C, formation of NaAl_7_O_11_ impurities disrupted the morphology. The crystal flowers form through a two-stage process involving anisotropic lamellar growth followed by spatial agglomeration and assembly. Specifically, selective adsorption of SO_4_^2−^ on the α–Al_2_O_3_ (0001) basal plane suppresses axial growth, while numerous nuclei agglomerate and stack in different orientations to form a radial three-dimensional flower-like architecture. Figure 6a,b illustrate this mechanism. Three-dimensional hierarchical/porous structures can also be regulated through dissolution-precipitation kinetics, which depend on precursor dissolution, diffusion, and competition between nucleation and growth, as well as morphology-dependent crystallization associated with high surface-to-volume ratios, near-surface lattice relaxation, and coordinatively unsaturated dipole centers. Fu et al. [96] prepared γ–Al_2_O_3_-based composite microspheres from γ–AlOOH yolk-shell precursors (Figure 6c) by Fe impregnation and carbon source-assisted annealing. The product consisted mainly of γ–Al_2_O_3_@Fe_3_O_4_@C at 500–600 °C and transformed to γ–Al_2_O_3_@FeAl_2_O_4_@Fe@C at 700–800 °C [96]. This temperature-dependent multiphase yolk-shell system provides a model for magnetic/dielectric component coupling and interfacial design (Figure 6d–f), as do N,P co-doped Fe_3_O_4_@C hollow shell–core heterostructures [97]; neither is a direct product of the six selected precursors.

Overall, the three strategies describe morphological evolution from the complementary perspectives of interfacial chemistry, mass transfer, and surface physics. Current studies on the three-dimensional hierarchical assembly of aluminosilicate mineral-based ceramics are primarily based on alumina systems. Nevertheless, the effects of impurity ions and mineral phase-transformation pathways on assembly behavior are not yet well understood, and the relationship between EMW absorption performance and hierarchical structure remains poorly established. Additional in situ characterization and multiscale simulations are needed to clarify assembly dynamics and examine structural designs aimed at impedance compatibility. Performance implication: porosity primarily reduces the effective permittivity and improves impedance compatibility, although quantitative links between pore parameters and RL_min_/EAB are still limited (Table S1).

## 4. Phase Reconstruction of Aluminosilicate-Derived Ceramics and Effects of Impurity Chemistry on the Electromagnetic Response Mechanisms

The phase and morphological features described in Section 3 do not by themselves produce strong absorption; rather, they control the density, connectivity, and chemical environment of loss sites. This section links these structural features to dielectric loss, magnetic loss, and stability at high temperatures. Table S1 compiles RL_min_, EAB, sample thickness, frequency range, and measurement conditions for representative systems related to the six precursors. Because the reported test conditions differ, these values are used for quantitative trend comparison rather than direct ranking. Model and analogous systems used to explain general loss mechanisms and behavior at high temperatures [98,99,100,101,102] are summarized in Table S2.

The previous sections discussed phase-structure differentiation pathways and regulation strategies for the six aluminosilicate precursors. The discussion below focuses on how precursor impurity chemistry and structural features affect dielectric loss, magnetic loss, and service stability at high temperatures.

### 4.1. The Influence of Aluminosilicate-Derived Systems on Dielectric Loss

In the studies cited here, individual loss mechanisms are generally inferred from interpretation rather than separated directly by experiment, unless a study explicitly isolates a specific contribution.

**(1) Fundamental mechanisms of interfacial polarization and dipole relaxation**

In the GHz range, the imaginary permittivity ε″ of aluminosilicate-derived multiphase ceramics comprises polarization loss (ε_p_″) and conduction loss (ε_c_″). MSS-derived porous ceramics contain numerous interfaces, including grain boundaries, crystal/amorphous interfaces, ceramic/carbon contacts, and solid–gas pore walls [103,104,105,106,107]. These interfaces are commonly interpreted as sites for space-charge accumulation and relaxation, although their individual contributions are seldom quantified experimentally. Dipole polarization is generally associated with oxygen vacancies, coordinatively unsaturated Al^3+^ species, including five-coordinate intermediates, and impurity substitution [21]. Liu et al. [21] described morphology-dependent phase transformations and defect evolution in Al_2_O_3_/mullite systems. As conductive phases approach the percolation threshold, electron migration and short-range hopping may increasingly contribute to conduction loss; however, most studies have not experimentally separated conduction loss from polarization loss.

**(2) Relationship between morphology and dielectric response**

**Figure 7:**
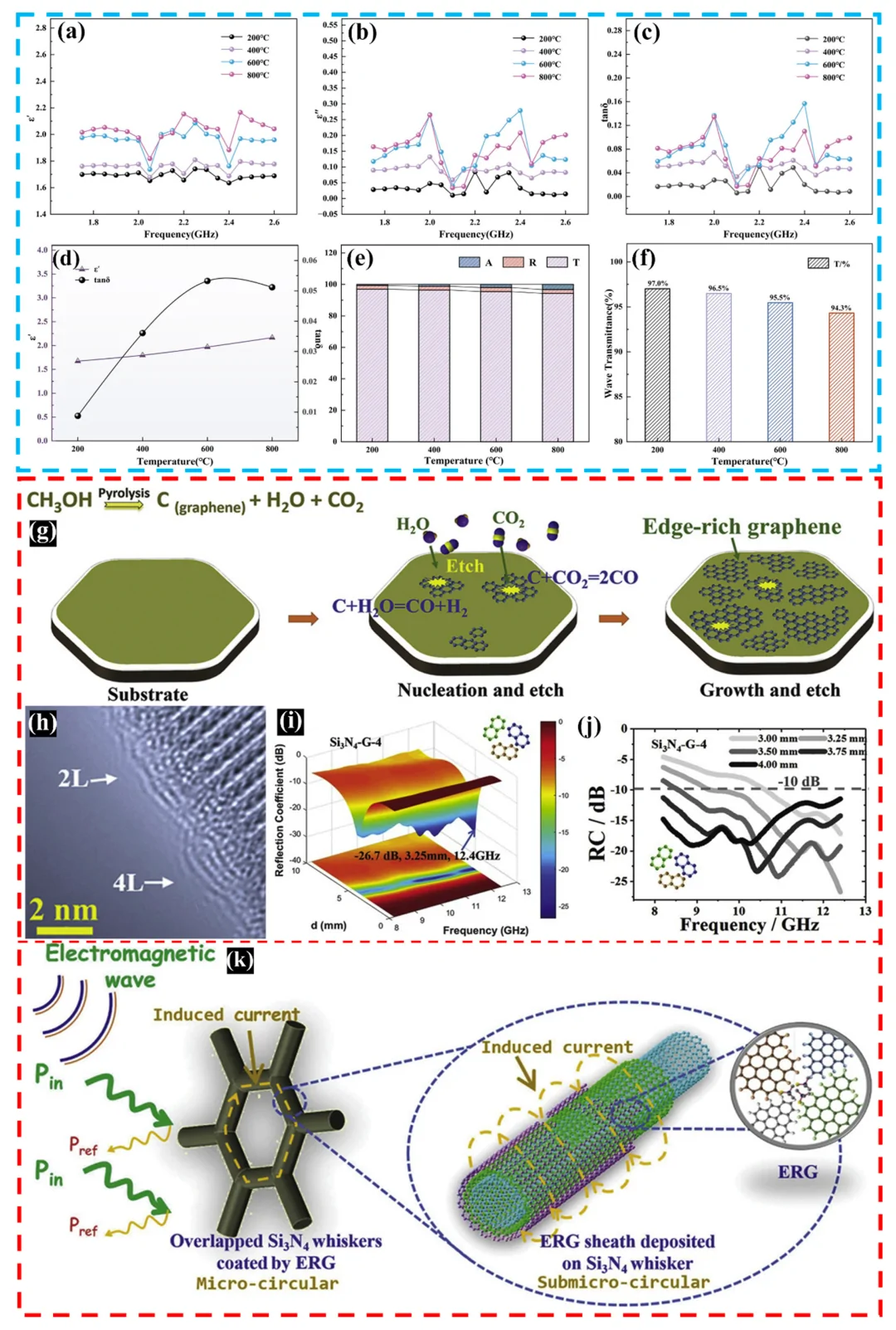
Microstructure–dielectric property relationship in aluminosilicate-derived ceramics. (**a**) real and (**b**) imaginary parts of the dielectric constant, (**c**) dielectric loss tangent (1.72–2.45 GHz), (**d**) dielectric constant and dielectric loss tangent value (2.45 GHz), (**e**) power loss coefficient, reflection coefficient, and transmission coefficient, (**f**) wave transmittance (2.45 GHz) of porous mullite ceramics at different temperatures. Reprinted with permission from ref. [108]. Copyright 2025 the American Ceramic Society. (**g**) Scheme showing the synthetic process, (**h**) TEM image, (**i**) 3D RL value, (**j**) reflection coefficients at different thicknesses, and (**k**) scheme showing the EMW absorption mechanisms of ERG/Si_3_N_4_ hybrids. Reprinted with permission from ref. [110,111]. Copyright Weinheim and 2021 Elsevier Ltd.

Microstructural features influence dielectric behavior through both polarization relaxation and conductive pathways [108,109,110,111,112]. (1) Pore architecture. Liu et al. [108] fabricated porous mullite ceramics with 70.5–74.9% porosity, where the optimized sample (74.9% porosity, 70 wt% Al_2_O_3_) exhibited an ultralow $\varepsilon^{\prime }$ of 1.73 and a tanδ of 2.0 × 10^−3^ at 2.45 GHz. The temperature dependence of these dielectric parameters, together with the energy-loss distribution and the wave transmittance of the same ceramics, is summarized in Figure 7a–f. Pores introduce solid–gas interfaces and reduce effective permittivity, while charge accumulation at pore walls is expected to contribute to ε_p_″. (2) Grain morphology. Fine grains can increase grain-boundary area and strengthen interface polarization [109], whereas anisotropic grains broaden the distribution of relaxation times. Mullite whiskers themselves have intrinsically low loss; when incorporated as a second phase, their orientation and interfacial bonding affect the dielectric response. (3) Phase distribution. Connectivity among crystalline, amorphous, and conductive phases determines heterointerface density and the relative contributions of ε_p_″ and ε_c_″. Ye et al. [110] grew edge-rich graphene within porous Si_3_N_4_ ceramics and achieved strong absorption; although this is a non-aluminosilicate analog, it illustrates how a conductive phase can be coupled with a porous ceramic skeleton. Figure 7g–k shows the corresponding synthesis scheme, microstructure, reflection-loss maps, and attenuation mechanism of this system [110,111]. In most studies, polarization and conduction losses are inferred rather than directly separated.

**(3) Intrinsic dielectric loss characteristics of different precursors**

(1) Kaolin. Pure, dry kaolin has intrinsically low dielectric loss, although the measured loss varies with purity, moisture, associated minerals, frequency, temperature, modification, and sintering conditions [113]. Impurities can increase dielectric loss. (2) Sillimanite. Owing to its crystal structure and wide bandgap [114], sillimanite is a favorable low-loss microwave dielectric [79]; associated ZrO_2_ may remain as a higher-permittivity second phase [115]. (3) Perlite. Typical perlite contains about 0.5–1.5 wt% Fe_2_O_3_ together with alkali-metal oxides, but the cited studies do not establish that Fe enters mullite or forms nanoscale Fe_3_O_4_ in situ. (4) Fly ash and coal gangue. Their absorption behavior is strongly influenced by unburned carbon and conductive loss, and the carbon content of coal gangue varies more widely than that of fly ash. (5) Red mud. Carbothermal reduction can convert Fe_2_O_3_ to Fe_3_O_4_ and subsequently to α-Fe/(Fe, C), generating conductive or metallic phases and additional heterointerfaces that may increase dielectric and magnetic losses [28,29,30]. The resulting contribution depends on Fe-phase composition, oxidation state, particle size, spatial distribution, and measurement conditions; total Fe_2_O_3_ content alone is therefore not a reliable predictor of dielectric loss. Hematite and goethite may also contribute polarization and conduction losses, but these effects must be evaluated together with those of reduced Fe phases.

### 4.2. Effects of Aluminosilicate-Derived Systems on Magnetic Loss

Pure Al_2_O_3_ and mullite generally lack a distinct magnetic-loss mechanism, and the introduction of a magnetic component can broaden the available attenuation pathways. The total iron contents of the six aluminosilicate precursors vary widely, but the magnetic behavior is governed primarily by iron valence, crystal phase, particle size, magnetic-domain state, temperature, and compatibility with dielectric loss. Magnetic response therefore cannot be ranked solely according to Fe_2_O_3_ mass fraction. Studies of N,P co-doped Fe_3_O_4_@C hollow shell–core structures [97], single-component Fe_3_O_4_ yolk-shell architectures [116], ferrite/carbon composites [117], and Fe_3_O_4_@C@PPy double-shell systems [118] indicate that magnetic loss, dielectric loss, interfacial polarization, and impedance matching must be analyzed together. These Fe_3_O_4_, ferrite, and carbon composites serve as supporting examples of magnetic/dielectric coupling; they are not derived from the six selected precursors.

**(1) Low–iron system: perlite**

Direct evidence regarding the chemical state of low-concentration iron in perlite and its heat-treatment products. Whether Fe_3_O_4_ forms under carbothermal conditions, whether the product remains nanoscale, and whether its volume fraction is sufficient to generate a measurable magnetic behavior requires verification using XPS, TEM, magnetic characterization, and complex-permeability measurements. The expanded perlite/magnetic-separation fly ash cement composites reported by Wang et al. [119] exhibited favorable electromagnetic-wave absorption in a 2–18 GHz arch-method test. Quantitative EMW absorption data for perlite-derived ceramics are still limited; current evidence primarily concerns structure, dielectric behavior, and cement-based composites.

**(2) High-carbon iron-containing systems: fly ash and coal gangue**

Fly ash- and coal gangue-derived absorbing materials have been studied extensively, and performance is strongly dependent on composition [26,27,120,121]. Li et al. [26], for example, treated fly ash, coal gangue, and coal in Ar at a ratio of 1:1:1 to obtain an Fe_x_Si_y_/SiC/C multiphase composite with an *RL*_min_ of −30.9 dB. A separate study produced Fe_3_Si/SiC/C composites by adding exogenous ferric nitrate and obtained an *RL*_min_ of −47.1 dB [27]. FA/Co composites with externally loaded Co, produced by Yang et al. [121] and tested using the coaxial method, exhibited an *RL*_min_ of −44.20 dB at 17.58 GHz and a thickness of 1.34 mm, together with a maximum EAB of 5.13 GHz at 1.63 mm.

**(3) Fully endogenous magnetic system: red mud**

**Figure 8:**
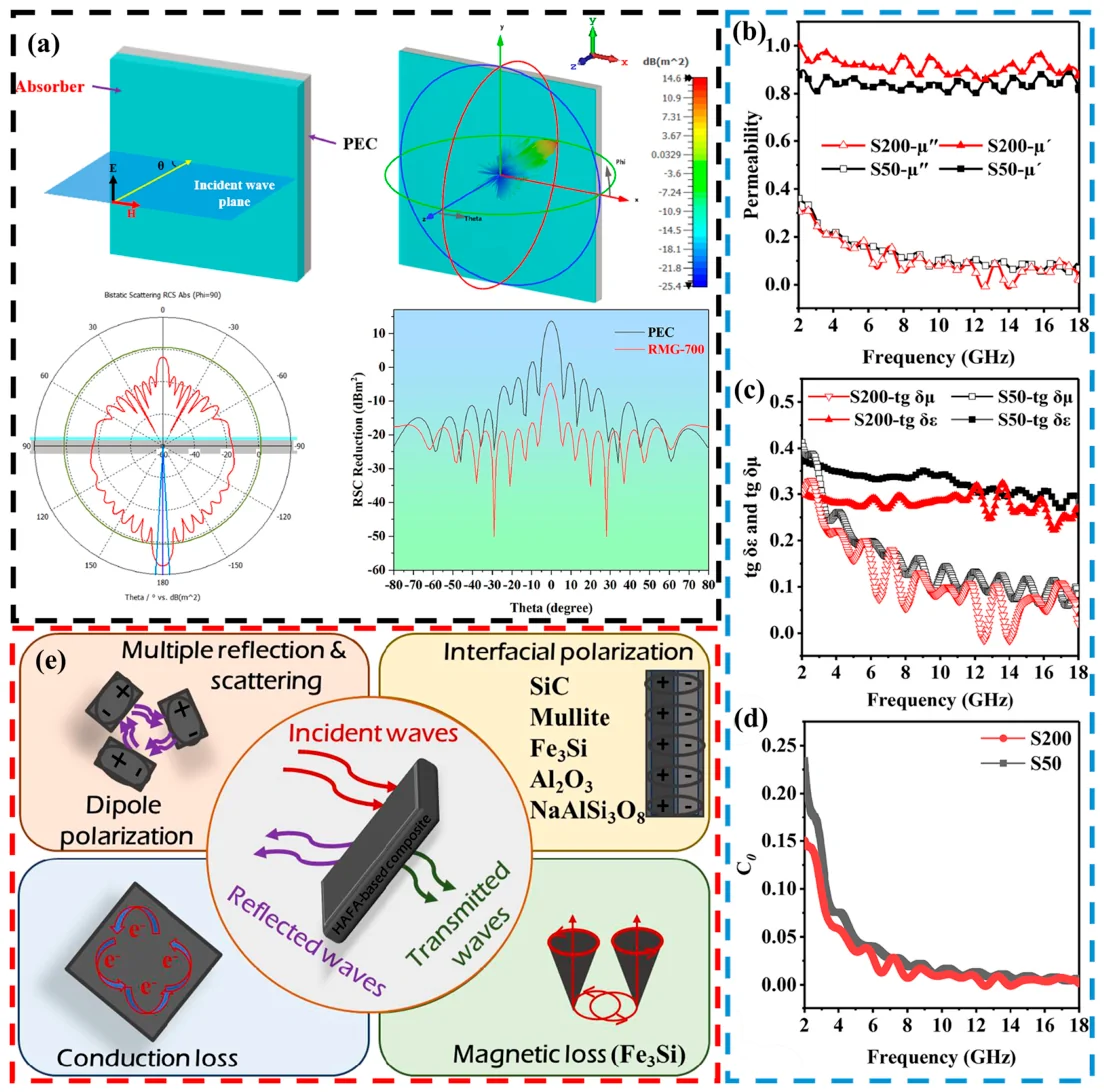
Magnetic loss in aluminosilicate-derived ceramics. (**a**) Schematic of the RCS simulation model and simulated RCS results for a PEC substrate coated with a 2.5 mm-thick RMG-700 layer at 11.1 GHz. Reprinted with permission from ref. [30]. Copyright 2026 Elsevier Ltd. (**b**) Permeability, (**c**) Dielectric and magnetic loss tangents, (**d**) Eddy current coefficient, (**e**) EMW absorption mechanism for S50 and S200 composites. Reprinted with permission from ref. [81]. Copyright 2026 Elsevier Ltd.

The red mud/coal gangue mixture constitutes a fully endogenous magnetic system in which calcination temperature controls iron-phase evolution and EMW absorption behavior [30]. The RMG–700 sample prepared at 700 °C contains Fe_3_O_4_ as the dominant magnetic phase and exhibits an *RL*_min_ of −34.3 dB at 4.4 GHz and 5.5 mm, with a maximum EAB of 3.52 GHz at 2.5 mm. At 900 °C, α–Fe and (Fe, C) alloy signals appear; the corresponding sample shows an *RL*_min_ of −18.2 dB at 12.56 GHz and 1.5 mm and an EAB of 3.44 GHz over 11.12–14.56 GHz at 1.5 mm. Figure 8a presents the associated RCS simulation model and PEC-substrate results [30]. These data show that calcination temperature changes both phase assemblage and the impedance-matching window. Besides endogenous iron, unburned carbon, transition metals, and alkali-metal components can also reconstruct during high-temperature treatment, altering interfacial chemistry and conductive pathways in solid waste-derived materials. In reported fly ash and red mud carbothermal systems, iron-based particles, carbon phases, and multiphase ceramic matrices form abundant heterointerfaces that may support space-charge accumulation, dipole relaxation, and conductive loss under alternating electromagnetic fields [81]. Figure 8b–e summarizes the electromagnetic parameters, eddy current coefficients, and proposed attenuation mechanisms for these fly ash-based absorbers. Nevertheless, the relative contribution of each mechanism still requires verification using frequency- and temperature-dependent dielectric spectroscopy, complex-permeability measurements, and experimental separation of conduction and polarization losses. Reflection loss is jointly controlled by dielectric and magnetic responses as well as phase composition, particle size, filling ratio, frequency, and matching thickness. The transition from Fe_3_O_4_ to α-Fe/(Fe, C) further indicates that iron-phase identity, rather than total iron content, governs the matching window and magnetic/dielectric response [30].

### 4.3. High-Temperature Dielectric and Oxidation-Resistant Service Stability of Aluminosilicate-Derived Systems

**Figure 9:**
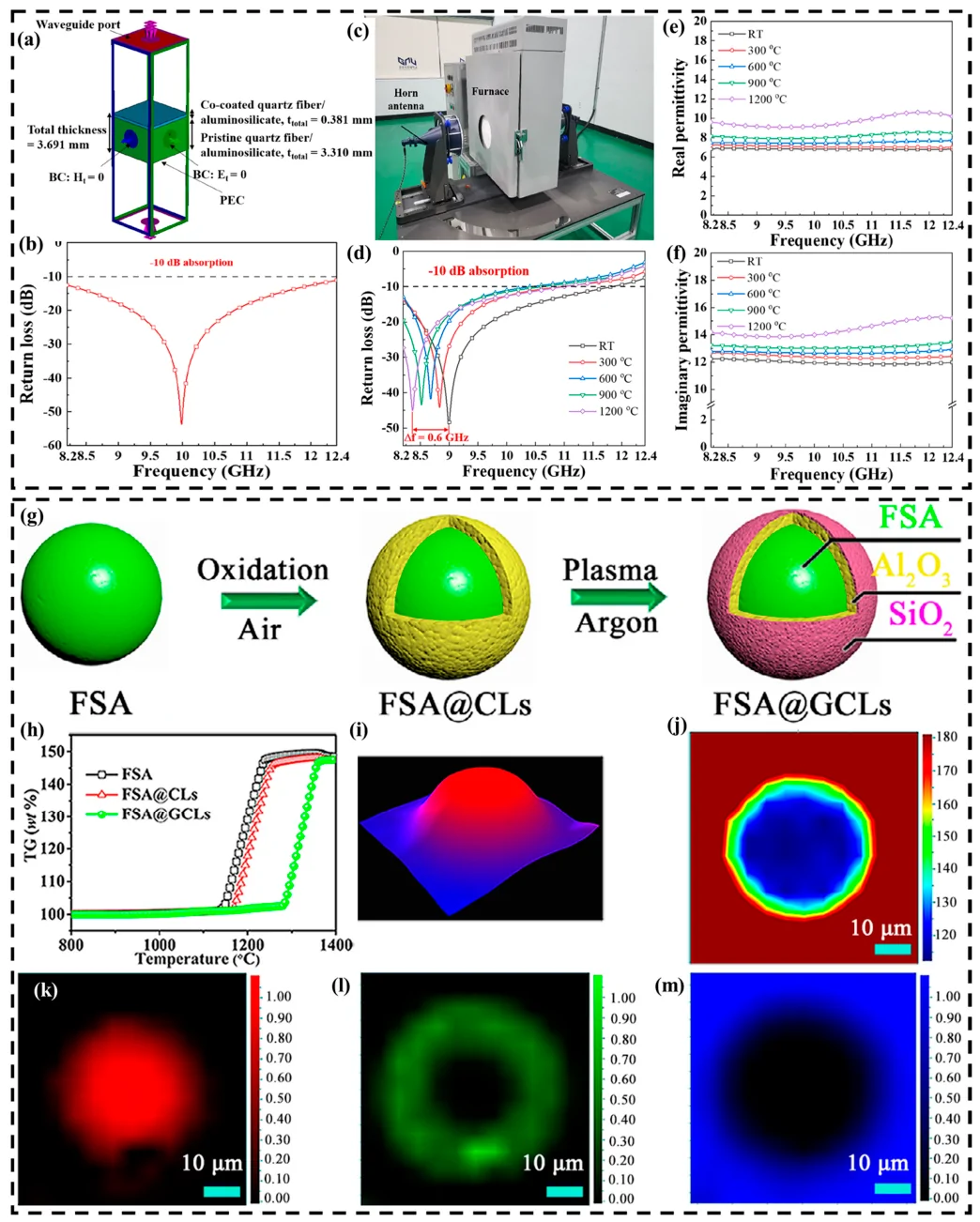
EMW absorption of aluminosilicate-derived ceramics in complex environments. (**a**) Electromagnetic simulation model of proposed high-temperature radar-absorbing composite with boundary conditions and (**b**) microwave absorption performance of the designed high-temperature microwave absorber; (**c**) Photograph of the free-space measurement system linked to a high-temperature furnace; (**d**) Measured absorption performance of proposed high-temperature radar-absorbing composite at various temperatures; (**e**) Real and (**f**) imaginary permittivity components of the Co-coated quartz fiber/aluminosilicate composite measured at various temperatures. Reprinted with permission from ref. [127]. Copyright 2022 Elsevier Ltd. and Techna Group S.r.l. (**g**) Schematic illustration of the synthesis process of FSA@GCLs and three-dimensional structure of FSA@GCLs. (**h**) TG of FSA, FSA@CLs and FSA@GCLs from 30 °C to 1400 °C; (**i**) Confocal Raman 3D mapping of FSA@GCLs. Schematic illustration of 2D mappings corresponding to (**j**) the mixture of (**k**) FSA, (**l**) Al_2_O_3_ and (**m**) SiO_2_ at 700 °C in air. Reprinted with permission from ref. [130]. Copyright 2019 Elsevier B.V.

A major advantage of ceramic-based absorbers over polymers and magnetic metals is their ability to operate at elevated temperatures. Tang et al. [122] reviewed high-temperature-resistant ceramic electromagnetic absorbers and emphasized that adjustable dielectric constants, high strength, and oxidation resistance make ceramics attractive for such applications. Xia et al. [123] similarly reviewed high-temperature microwave absorbers and discussed the temperature and frequency dependence of dielectric and magnetic losses. Because electromagnetic parameters vary with temperature, maintaining impedance matching during high-temperature service remains difficult. Beyond thermal challenges alone, practical harsh environments frequently impose coupled demands of oxidation barrier capabilities, structural durability, and corrosion resistance. To address these multi-environmental threats, ceramic surface encapsulation has emerged as an effective protection paradigm. For instance, atomic layer deposition of conformal oxide shells onto magnetic alloys, as demonstrated in FeSiAl@ZnO@Al_2_O_3_ core–shell particles [124], provides an impermeable physical barrier against corrosive media in salt-fog environments while maintaining strong low-frequency attenuation. Under elevated-temperature service, ceramic networks further serve to stabilize dielectric properties and prevent core oxidation; systems such as multiscale dielectric architectures [125], La_0.5_Sr_0.5_CoO_3_/Al_2_O_3_ coatings with thermally responsive permittivity [126], and coated quartz-fiber/aluminosilicate composites [127] have been explored to suppress high-temperature impedance degradation. For aluminosilicate matrices, kaolin-derived mullite composites reinforced with fly ash cenospheres retain high compressive strength after exposure to 800 °C [128]. Nevertheless, among these protective designs, in situ high-temperature electromagnetic evaluations remain sparse; to date, only Choi et al. [127] have systematically tracked dynamic X-band permittivity and permeability transitions from room temperature up to 1200 °C. Figure 9a–f summarize the electromagnetic simulation model, absorption performance, test-system images, and electromagnetic parameters of the cobalt-coated quartz fiber/aluminosilicate composite. Post-heat-treatment measurements and theoretical calculations in other studies cannot be considered equivalent to in situ high-temperature absorption measurements because a material may retain its phase assemblage after cooling while undergoing reversible changes in conductivity, polarization, and magnetic response during heating. High-temperature in situ dielectric spectra, complex permeability, and thermal-cycling data are still scarce for different precursor systems tested under comparable conditions. Stability at high temperatures should therefore be regarded as a property that requires direct experimental verification rather than inference from material class. These examples are model or protective systems rather than direct products of the six precursors; the Co-coated quartz fiber/aluminosilicate composite remains the only example with in situ X-band data extending from room temperature to 1200 °C.

Performance degradation at high temperatures is governed by impurity chemistry. The total Na_2_O + K_2_O content of typical expanded perlite can reach ~10 wt% [80]. In such alkali-rich aluminosilicate systems, thermally activated migration of alkali-metal ions (Na^+^, K^+^) along open framework channels at elevated temperatures is expected to increase conduction loss and cause drift in permittivity, by analogy with the ion-migration behaviour documented in other ionic solids [129]. Although systematic high-temperature dielectric spectroscopy for perlite-derived ceramics remains limited, evaluating these ionic transport mechanisms is essential because excessive ion conduction at elevated temperatures inevitably deteriorates impedance matching. Kaolin and sillimanite commonly contain lower concentrations of alkali-metal and iron impurities, but this observation does not show that they have superior electromagnetic stability at 600–1000 °C. The mullitization-temperature range of sillimanite also changes with holding time, particle size, impurity composition, and formulation. Fe_3_O_4_, Fe, and carbon phases in red mud- and coal gangue-derived systems may experience oxidation or ablation in high-temperature oxygen-containing environments, thereby resulting in changes in dielectric and magnetic behaviors. The compositions of fly ash, coal gangue, and red mud vary with source and processing conditions, and these compositional fluctuations can directly affect the performance at high temperatures of large-scale products [28,44].

In oxygen-containing high-temperature conditions, carbon-phase ablation and deep oxidation of iron-based phases are likely the principal degradation channels in solid waste-derived systems, although direct in situ evidence for the six precursor systems is limited. Dense ceramic layers can function as oxygen barriers and improve resistance to oxidation. As shown in Figure 9g–m, Guo et al. [130] formed Al_2_O_3_/SiO_2_ gradient shells on FeSiAl particles and showed their effect on oxidation kinetics. The gradient structure reduces abrupt mismatches in thermal expansion and composition between the magnetic core and ceramic shell. The temperature of 1279 °C measured by TG/DSC marks only the onset of rapid oxidation and is not an in situ absorption-test temperature. This work offers a useful oxidation-protection model, but coating continuity, shell thickness, thermal-expansion compatibility, and thermal-cycling resistance still need to be considered. This FeSiAl-based core–shell system is an oxidation-protection model, not a direct six-precursor-derived ceramic.

EMW absorption in aluminosilicate mineral-based ceramics is determined by precursor composition, phase-evolution pathway, and microstructural features. Kaolin- and sillimanite-based matrices are generally interpreted as defect-dipole and grain-boundary polarization systems, whereas fly ash and coal gangue often depend on carbon networks for dielectric loss. Red mud-derived systems can generate tunable dielectric and magnetic losses through iron-phase evolution, although the magnitude and dominant attenuation mechanisms vary with Fe-phase composition and measurement conditions [28,29,30]. Total iron content alone does not determine magnetic loss; iron valence, crystal phase, particle size, and spatial distribution are also critical. Under high-temperature oxidizing conditions, carbon ablation and iron-phase oxidation are expected to be major degradation pathways, but direct service-condition evidence remains limited. Alkali-ion migration in perlite may additionally cause dielectric drift. Future research should integrate in situ high-temperature testing with quantitative correlations among electromagnetic loss, phase assemblage, impurity chemistry, pore architecture, and precursor characteristics.

## 5. Conclusions and Prospects

### 5.1. Conclusions

Aluminosilicate mineral-based ceramics show considerable promise for high-temperature EMW absorption, although the six precursors cannot yet be directly ranked because reported studies use different measurement conditions and most high-temperature data are derived from model systems. Kaolin and sillimanite mainly provide low-loss ceramic skeletons, whereas fly ash, coal gangue, and red mud can introduce carbon-, silicide-, and iron-oxide-based loss phases. The principal unresolved issues include in situ high-temperature electromagnetic characterization, oxidation stability of carbon/Fe phases, and quantitative structure–performance relationships.

### 5.2. Key Challenges

Three major challenges remain. First, feedstock variability reduces batch-to-batch reproducibility and limits transferability of processing conditions. Second, the absence of in situ high-temperature electromagnetic characterization prevents direct verification of how loss mechanisms evolve during service. Third, quantitative relationships linking porosity, phase distribution, impurity chemistry, electromagnetic parameters, oxidation behavior, and long-term performance remain insufficiently established.

### 5.3. Future Development Directions

Future research should prioritize: (1) machine-learning-assisted prediction of composition–process–performance relationships while accounting for feedstock variability; (2) wideband in situ electromagnetic testing at high temperatures; (3) oxygen-blocking microstructures that protect conductive and magnetic phases; and (4) phase-field/finite-element modeling of anisotropic crystal growth, impurity segregation, and dielectric relaxation.

## Data Availability

No new data were created or analyzed in this study. Data sharing is not applicable to this article.

## Supplementary Materials

The following supporting information can be downloaded at: https://www.mdpi.com/article/10.3390/nano16191245/s1, Table S1: Comparative electromagnetic-wave absorption data for representative systems associated with the six selected precursors; Table S2: Model/analogous systems cited for general loss mechanisms and high-temperature behavior.
