# Supplementary material for: Research Progress in Phase–Structure Evolution and Electro-magnetic–Wave Absorption Performance of Aluminosilicate Mineral–Based Ceramics
Source: Nanomaterials (Basel). 2026 Oct 1;16(19):1245. doi: 10.3390/nano16191245 (PMC13666749; doi:10.3390/nano16191245)
Supplement: Supplementary file 1 [file nanomaterials-16-01245-s001.zip › nanomaterials-4543258-supplementary.pdf]

Research Progress in Phase–Structure Evolution and Electro-magnetic–Wave Absorption Performance of Aluminosilicate Mineral–Based Ceramics

Zhongyi Bai[1*], Qichen Zhang[1], Song Li[1], Chunjiang Li[1], Biao Zhao[2*], Mengjie Wang[1], Xuwen Li[1], Rong Wang[1], Xiaoqin Guo[1*]

[1] Henan Key Laboratory of Aeronautical Materials and Application Technology, School of Materials Science and Engineering, Zhengzhou University of Aeronautics, Zhengzhou, Henan, 450046, China.

[2] School of Materials Science and Engineering, Zhengzhou University, Zhengzhou, Henan, 450001, China.

Corresponding author:

Dr. Zhongyi Bai, E–mail address: zhy_bai@zua.edu.cn

* Prof. Biao Zhao, E–mail address: zhaobiao1813@163.com

* Prof. Xiaoqin Guo, E–mail: guoxq@zua.edu.cn

Table S1. Comparative electromagnetic-wave absorption data for representative systems associated with the six selected precursors.

Precursor	Representative final phase/system	Frequency range/method	Sample/thickness (mm)	RL_{min} (dB)	EAB (GHz)	ε′/ε″; μ′/μ″	Measurement temperature	Ref.
**Kaolin**	Graphite/geopolymer composite (Gr/GP = 12:1)	2–18 GHz/Coaxial	Bulk/4.15	−64.8 (5.1 GHz)	1.9 (4.2–6.1)	ε′:15.0–9.5,ε″:4.8–3.5 μ′ ≈ 1.1,μ″ ≈ 0	RT (As-cured)	[78]
	Graphite/geopolymer composite (Gr/GP = 9:1, post-treated)		Bulk/3.64	−55.4 (6.4 GHz)	2.3 (5.3–7.6)	ε′:9.0–5.5,ε″:2.5–1.0 μ′ ≈ 1.1,μ″ ≈ 0	RT (After 600 °C N_2 treatment)	
**Sillimanite**	Poorly densified bulk Al_2SiO_5 (Conventional sintering)	~16 GHz/Hakki-Coleman	Bulk cylinder/11ϕx5.4	NR	NR	ε_r = 4.43 $\tan\delta \approx$ 3.8x10^{-4}	RT (Sintered at 1525 °C)	[79]
**Perlite**	Expanded perlite (40 vol.%, 1 mm) + 3 wt% Carbon Black/Gypsum	2–18 GHz/Arch reflection & Waveguide(ε)	Bulk/20.0	−15.0 (2.0 GHz)	12.5 (2–3, 5–6.5, 8–18)	ε′ :3.0 – 3.7 ε″: 0.8 – 2.1 μ_r = 1	RT	[80]
**Fly ash**	High-alumina fly ash S200: mullite/Al_2O_3/SiC-containing composite	2–18 GHz/Coaxial	Powder ring /3.0	−58.82 (7.88 GHz)	2.75	ε/μ reported as curves	RT	[81]
**Coal gangue**	Fe_xSi_y/SiC/C from fly ash + coal gangue + coal	2–18 GHz/Coaxial	Powder ring /1.5 & 2.5	−30.9 (8.4 GHz, d = 2.5mm)	3.6 (12.4–16.0, d = 1.5mm)	ε/μ reported as curves	RT	[26]
**Red mud**	RMG-900 α-Fe/(Fe, C) composite	2–18 GHz/Coaxial	Powder ring/1.5	−18.2 (12.56 GHz)	3.44 (11.12–14.56)	ε′: 23.2 – 13.5 ε″: 6.5 – 9.6 μ′: 1.085 – 0.986	RT (Calcined at 900 °C)	[30]
	RMG-700 Fe_3O_4 composite		Powder ring/5.5	−34.3 (4.4 GHz)	Reported as curves	ε′ :11.9 – 7.2 μ′ :1.089 – 1.068	RT (Calcined at 700 °C)	

NR = Not Reported;

RT = Room Temperature;

Fig. = Only frequency-dependent curves are reported.

Values were compiled from studies conducted under different measurement conditions and are intended for trend comparison rather than ranking.

Table S2. Model / analogous systems cited for general loss mechanisms and high-temperature behavior.

Precursor	Representative final phase/system	Frequency range/method	Sample/thickness (mm)	RL_{min} (dB)	EAB (GHz)	ε′/ε″; μ′/μ″	Measurement temperature	Ref.
**SiC_f/SiC/Mu with SiC interphase**	PIP-derived SiC interphase (5 wt% PCS) in SiC_f/Mu	X–band/Waveguide	Bulk/2.6	−17.7 (10.0 GHz)	3.0	ε′ ≈ 6.2, ε″ ≈ 2.4;	RT	[98]
					3.3	ε′:9.7–8.8, ε″: 12.3–11.6	700 °C	
**SiC fiber/mullite matrix composite**	SiCf/Mu	X–band/Waveguide	Bulk/2.9 & 3.4	−38 (12.0 GHz, d = 2.9mm)	4.2 (8.2–12.4, d = 3.4mm)	ε′:5.7–5.3, ε″:3.6–2.6 μ_r = 1	RT	[99]
**SiC_f/Mu + SiC/Ti_3SiC_2 fillers**	Hybrid SiC/Ti_3SiC_2 in SiC_f/ Mu	X–band/Waveguide	Bulk/3.5	−53 (8.64 GHz)	2.1(8.20–10.30)	ε′:6.79–6.66, ε″:3.25–2.45 μ_r = 1	RT	[100]
**Mullite/SiB_6 APS coating**	MS15: 15 wt% SiB_6 self-healing coating (As-sprayed)	X–band/Waveguide	Bulk/1.8	−34.6 (9.48 GHz)	2.09 (8.48–10.57)	ε′: 20.3–19.2, ε″: 5.2–5.3 μ_r = 1	RT	[101]
	MS15-600: Annealed at 600 °C in air for 5 h			−28.62 (11.10 GHz)	2.07 (10.05–12.12)	ε′: 19.5–17.8, ε″: 5.0–5.1 μ_r = 1	RT	
	MS15-800: Annealed at 800 °C in air for 5 h			−23.81 (10.63 GHz)	2.35 (9.47–11.82)	ε′: 16.5–15.3, ε″: 4.5–4.3 μ_r = 1	RT	
**MXene/Ni/Aluminosilicate glass(AS)**	Binary $Ti_3C_2T_x$/AS mesoporous glass	2–18 GHz/Coaxial	Bulk/2.35	−41.65 (10.5 GHz)	2.6 (8.0–10.6)	ε′: 10.0–9.0, ε″: 2.0–1.8 μ′≈1.0, μ″≈0	RT	[102]
	Hierarchical $Ti_3C_2T_x$/Ni/AS		Bulk/2.0	−59.5 (10.8 GHz)	3.0 (8.2 – 11.2) 4.1 (12.4 – 16.5)	ε′: 15.0–11.0, ε″: 4.0–3.5 μ′≈1.0, μ″≈0		

References:

26. Li, G.; Li, K.; Hou, R.; Huang, Y.; Zhang, K.; Liang, L. Recycling of solid waste fly ash and coal gangue for fabricating low-cost and high-efficiency FexSiy/SiC/C composites as microwave absorbents. *J. Environ. Chem. Eng.* **2025**, 13, 117003.

30. Xue, X.; Liang, L.; Li, K.; Jia, K.; Zhang, K.; Li, G. A "Waste-to-Resource" strategy for fabricating efficient ceramic-based composite microwave absorbers. *J. Environ. Chem. Eng.* **2026**, 14, 122679.

78. Zhang, Y.; He, P.; Yuan, J.; Yang, C.; Jia, D.; Zhou, Y. Effects of graphite on the mechanical and microwave absorption properties of geopolymer based composites. *Ceram. Int.* **2017**, *43*, 2325-2332.

79. Induja, I.J.; Sebastian, M.T. Microwave dielectric properties of mineral sillimanite obtained by conventional and cold sintering process. *J. Eur. Ceram. Soc.* **2017**, *37*, 2143-2147.

80. Xie, S.; Ji, Z.; Yang, Y.; Hou, G.; Wang, J. Electromagnetic wave absorption enhancement of carbon black/gypsum based composites filled with expanded perlite. *Compos. Part B Eng.* **2016**, *106*, 10-19.

81. Salisu, I.; Wang, X.; Ma, S.; Ou, Y.; Hui, H.; Xu, L. High-alumina fly ash-derived microwave absorbents with enhanced electromagnetic wave attenuation via alkaline activation and carbothermal reduction. *Waste Manage.* **2026**, 214, 115412.

98. Gao, H.; Luo, F. Effect of SiC interphase on the mechanical, high-temperature dielectric and high-temperature microwave absorption properties of the

SiCf/SiC/Mu composites. *Ceram. Int.* **2022**, *48*, 18567-18578.

99. Gao, H.; Luo, F.; Wen, Q.; Qing, Y.; Zhou, W. Effect of preparation conditions on mechanical, dielectric and microwave absorption properties of SiC fiber/mullite matrix composite. *Ceram. Int.* **2019**, *45*, 11625-11632.

100. Gao, H.; Luo, F.; Nan, H.; Wen, Q.; Qing, Y.; Wang, C.; Zhou, W.; Zhu, D. Improved mechanical and microwave absorption properties of SiC fiber/mullite matrix composite using hybrid SiC/Ti_3SiC_2 fillers. *J. Alloys Compd.* **2019**, *791*, 51-59.

101. Feng, R.; Qing, Y.; Jiang, C.; Yang, J.; Cao, Y.; Zhang, L. Mullite/SiB6 coating for microcrack heal and microwave absorption performance at high temperatures. *J. Eur. Ceram. Soc.* **2026**, *46*, 117881.

102. Luo, W.; Wang, M.; Wang, K.; Yan, P.; Huang, J.; Gao, J.; Zhao, T.; Ding, Q.; et al. A robust hierarchical MXene/Ni/Aluminosilicate glass composite for high-performance microwave absorption. *Adv. Sci.* **2022**, *9*, 2104163.
